# Micro-Gap Weld Seam Contrast Enhancement via Phase Contrast Imaging

**DOI:** 10.3390/ma18061281

**Published:** 2025-03-14

**Authors:** Yanfang Yang, Yonglu Yang, Wenjun Shao

**Affiliations:** 1 School of Transportation and Logistics Engineering, Wuhan University of Technology, Wuhan 430063, China; yangyanfang@whut.edu.cn (Y.Y.); yyl301129@whut.edu.cn (Y.Y.); 2 Engineering Research Center for Logistics Technology and Equipments, Wuhan University of Technology, Wuhan 430063, China

**Keywords:** differential phase contrast, automatic welding, image contrast, welding seam detection

## Abstract

The precision and stability of seam position detection are critical for single-square-groove weld seams formed using two thin metal plates. However, traditional methods, such as structured laser light imaging, struggle with narrow seams that lack misalignment and have high reflectivity, while non-structured light approaches are prone to welding light interference and speckle noise. To overcome these challenges, we propose a versatile optical design that leverages differential illumination to generate differential phase contrast (DPC) images. By processing images captured under differential illumination, the DPC method notably enhances seam edge contrast and suppresses welding light noise, improving the detection robustness and reliability. This approach provides a promising solution for high-precision weld seam detection in challenging environments.

## 1. Introduction

**Figure 1:**
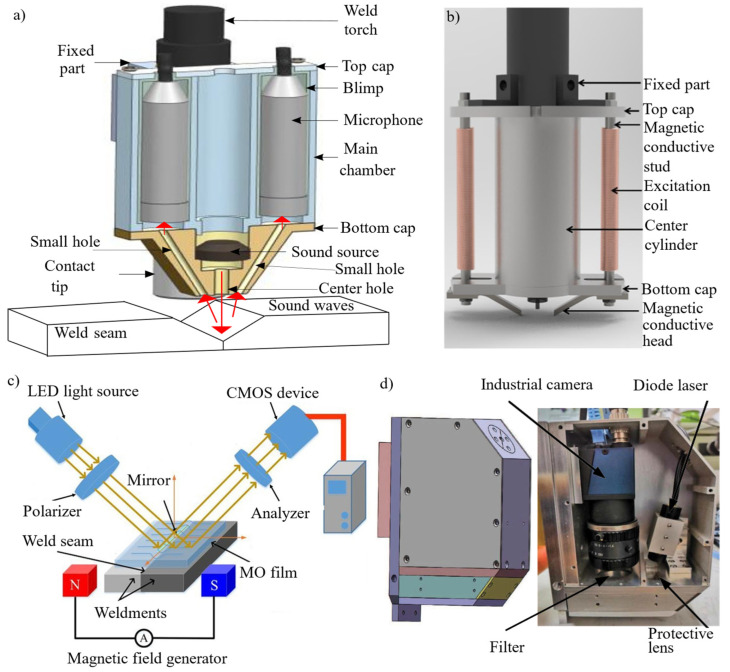
Representative state-of-the-art welding seam tracking technologies: (**a**) structure of a two-channel acoustic sensor for V-groove weld seam tracking [1]; (**b**) 3D model of a magnetically controlled arc sensor for detecting variable-gap seams [2]; (**c**) schematic of a magneto-optical imaging sensor for micro-gap weld seam tracking [3]; (**d**) real photograph of a laser triangulation sensor for spatial circular weld seam tracking [11].

Welding quality and productivity have significantly improved with the adoption of automatic seam detection and tracking systems. As shown in Figure 1, various types of seam detection sensors are available, including ultrasonic sensors [1], arc sensors [2], magneto-optical sensors [3], tactile sensors [4], and vision sensors [5,6,7,8,9,10,11]. Each method has its unique advantages for specific welding applications. Among these, vision sensors are widely preferred due to their high speed, precision, non-contact measurement capability, and ability to provide three-dimensional information about the weld seam.

Vision-based weld seam detection methods can be broadly classified based on the type of illumination. The first category employs structured light, typically using laser lines generated by a cylindrical lens [11,12,13,14,15,16,17,18,19,20,21,22,23,24,25,26]. The second category utilizes wide-field illumination, usually produced by a collimated beam from a condenser or convex lens [27,28,29]. Based on the light collection approach, these methods can further be divided into dark-field imaging [28,29], and bright-field imaging [27]. Due to the highly reflective surface of metal weldments, dark-field imaging is the preferred choice for most vision-based weld seam detection systems. Besides the above classifications, advancements in weld seam tracking systems have also been driven by the development of vision sensors. Optical coherence tomography (OCT), known for its three-dimensional imaging capability, has been applied in welding processes. Its coaxial imaging configuration allows the imaging light source and the welding light source to share the same optical path, enabling real-time capture of the weld profile within the welding pool [30,31].

Structured laser light-based weld seam detection is particularly effective for various weld groove types [32], including lap grooves [23], V-shaped grooves [11,14,17,33,34,35], U-shaped grooves [13], and tee-shaped grooves [15,16], as the laser line projection significantly bends at the seam location. However, for tight square grooves with seam widths smaller than 0.1 mm, the laser line often exhibits minimal shape deformation at the seam location, leading to unstable or failed detection [24,36].

To capture more surface features of the weld seam, non-structured illumination, such as wide-field illumination using light-emitting diodes (LEDs), has been employed for detecting tight square grooves. Although seam features are observable in the acquired images, their contrast is highly sensitive to factors like the surface texture, reflectivity, and flatness of the weldment. Poor seam contrast complicates image processing algorithms, making it challenging to achieve accurate and stable seam position extraction. To address these limitations, significant progress has been made in developing novel denoising optical sensors [27,28,29,37]. For example, combined structured and non-structured illumination demonstrated improved detection stability for tight square grooves [29]. Additionally, the seam image contrast was greatly enhanced by processing image series acquired under periodic wide-field illumination [37]. However, as multiple images are required to generate a single high-contrast image, the acquisition speed should be increased to meet the demands of high-speed welding. Beyond sensor optimization, researchers have explored advanced image processing methods to enhance the applicability of non-structured illumination for weld seam detection. For instance, an innovative Attention-Enhanced Feature Fusion Network (AFFNet) has been used to identify weld locations at the pixel level through enhanced feature detection [38]. The Kalman filter has been employed to predict and optimize seam detection results for tight square grooves under LED illumination [28]. To further improve seam position detection under challenging conditions, probabilistic and deep learning-based methods have been explored. A particle filter was employed to estimate the seam position by incorporating both current and past observations, enhancing robustness against strong light noise [39]. Although the particle filter improved the detection robustness under welding light, it did not enhance the detection accuracy because the seam edge contrast was compromised under welding light illumination. A generative adversarial network (GAN) was used to denoise weld fumes, enabling clearer seam detection [40]. To mitigate welding light noise, a deep learning model was developed for key point detection in fillet welding [41]. Furthermore, leveraging its strong noise resistance, a spatial–temporal deep learning model enabled direct seam tracking using molten pool image sequences [42].

As can be seen from the above analysis, tremendous progress has been made on improving the seam tracking accuracy under welding light noise. However, few studies have directly focused on improving the weld seam edge contrast using non-structured illumination methods from an optical design perspective. Building upon our previous research [37], we propose an improved optical design that incorporates the DPC imaging technique for seam detection in this study. DPC imaging has been demonstrated to enhance edge contrast in printed circuit board (PCB) inspection [43]. It has also gained widespread adoption in modern optical microscopy, where it increases the contrast of fine structures in biological specimens [44,45,46,47,48].

Unlike conventional imaging, which captures only the amplitude of light, the DPC method computationally recovers phase differences from two intensity images, allowing for the detection of features that are otherwise difficult to observe. Inspired by this approach, we designed an optical system that converts phase variations caused by the surface and edges of the weldment into intensity variations by calculating the difference between two intensity images.

To achieve real-time DPC imaging, we employ a high-speed LED controller and high-speed camera. A comparative analysis with existing seam detection methods demonstrates that the DPC image offers competitive results in terms of edge intensity and seam contrast. Additionally, since the DPC image is generated by subtracting one image from another, the welding light noise is inherently reduced, further enhancing detection robustness.

This research introduces several key contributions to vision-guided automatic welding processes. Firstly, the proposed DPC imaging method offers a novel optical design that notably improves the seam edge detection accuracy and robustness of existing seam tracking techniques. By capturing phase variations at the seam edge, the method enhances edge contrast, enabling more precise detection of the seam position and width, which is critical for high-precision laser welding applications. Moreover, the DPC method naturally reduces welding light noise, further enhancing detection robustness even in challenging, noisy conditions. The ability to capture phase variations, a feature that is not achievable with traditional seam tracking methods, forms the foundation for the enhanced seam contrast, making the proposed technique more effective under high-precision welding conditions. Additionally, the DPC imaging technique can be easily integrated into the existing vision sensors used in high-precision laser welding systems, such as robotic-arm laser welding machines. This integration has significant potential to improve the capability of current sensors to detect narrow-gap weld seams with higher accuracy, making it well suited for real-world industrial applications.

To the best of our knowledge, this is the first application of DPC imaging for real-time weld seam detection. With increased seam contrast and effective noise reduction, the seam extraction algorithm becomes simpler and more reliable.

## 2. Methods

### 2.1. Experimental Setup and Principle

**Figure 2:**
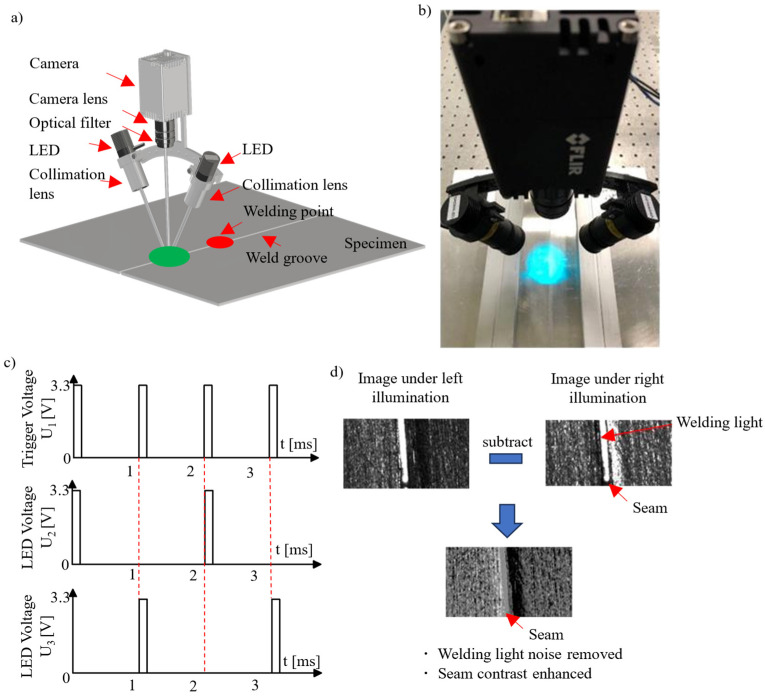
The experimental setup: (**a**) a three-dimensional model of the system, where red circle represents the welding point, and green circle represents the camera’s field of view; (**b**) an actual photograph of the system; (**c**) the control signal for the LEDs and camera, where U_1_ represents the control voltage for the camera, U_2_ represents the control voltage for the left LED, and U_3_ represents the control voltage for the right LED; (**d**) the concept of the proposed method.

A three-dimensional model and a photograph of our seam detection system for tight square grooves are shown in Figure 2a,b. The system primarily consists of the following components: a high-speed camera (FLIR: ORX-10GS, Wilsonville, OR, USA), camera lens (Navitar: NMV-50, Rochester, NY, USA), optical filter (Thorlabs: FBH470-10, Newton, MA, USA), blue LED (Thorlabs: M470L5, Newton, USA), high-speed LED controller (Thorlabs: LEDD1B, Newton, USA), condenser lens (Thorlabs: ACL2520U-A, Newton, USA), and analog output card.

The two LEDs emit light with the same spectrum and power, centered at 470 nm. The optical filter, also centered at 470 nm, effectively blocks most ambient and welding light. The condenser lens is used to collimate the LED light. The two high-speed LED controllers switch the LEDs on and off alternately, enabling sequential illumination of the workpiece. The LED driver can modulate the light at a maximum frequency of 5000 Hz. Synchronization between the LED driver and the camera is achieved via control signals from the analog output card, ensuring image acquisition when only one LED is active. The camera operates at a maximum frame rate of ~1000 Hz when the region of interest (ROI) is 200 pixels × 1000 pixels. The working distance is approximately 220 mm, yielding a spatial pixel resolution of 12 µm × 12 µm. Figure 2c illustrates the synchronization control signals for the LED and the camera.

The proposed method leverages a modern high-speed camera and high-speed LED driver, allowing the capture of right and left illumination images within ~2 ms, thereby minimizing motion artifacts during real-time weld seam detection. Figure 2d presents the concept and core idea of the proposed method, which enhances seam contrast through DPC imaging while removing spark noise from the acquired images. Spark noise is directly eliminated by calculating the difference between two images captured almost simultaneously. The mechanism for seam contrast enhancement is explained in detail in subsequent sections.

**Figure 3:**
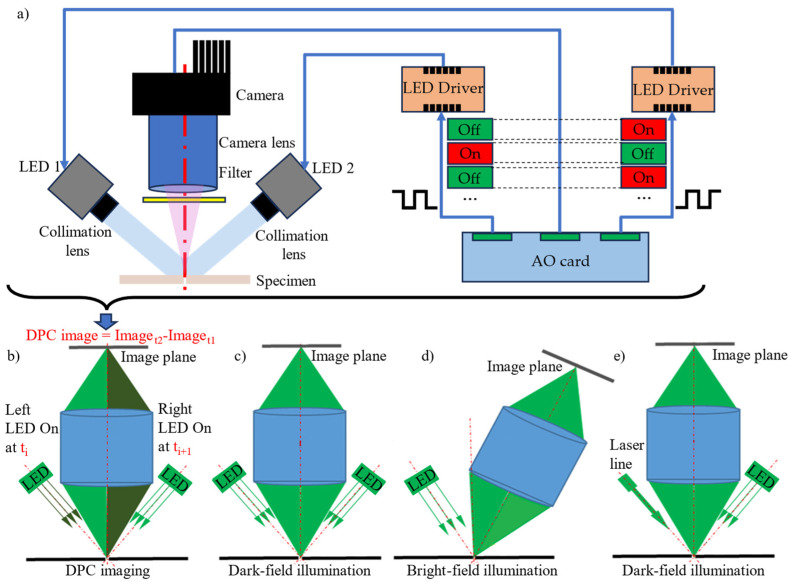
Schematics of the proposed DPC imaging method and comparisons with typical seam detection methods: (**a**,**b**) the proposed seam detection method based on DPC imaging; (**c**) a seam detection method with double-side illumination in dark-field mode; (**d**) a seam detection method with single-side illumination in bright-field mode; (**e**) a seam detection method combining structured light illumination and dark-field illumination.

Schematics of our setup are shown in Figure 3a,b. The angle between the optical axes of the two LEDs and the camera lens was designed to be larger than the light collection angle of the camera lens, ensuring that the camera collects diffused rather than reflected light. The system follows the principle of dark-field illumination to reduce specular reflections from the metal surface of the workpiece. As shown in Figure 3a, the acquisition of DPC images differs from traditional weld seam detection methods because the DPC image is obtained as the difference between images captured with alternating left and right side illumination. Comparing these images taken under different lighting conditions enables the extraction of phase information, which traditional seam detection methods cannot capture, thereby enhancing the details and contrast of the weld seam image. In biological specimens, phase changes in illumination light arise from variations in thickness and refractive indices. In our case, the phase changes result from height variations at the seam edge. Therefore, the proposed DPC imaging approach improves image contrast by converting height variations at the micro-seam edge into intensity variations displayed in the DPC image, making it particularly suitable for seam contrast enhancement in metal workpieces. According to the principle detailed in [45,48], the reflectance differential phase contrast can be calculated from two images under different illuminations using Equation (1):

(1) $$I_{DPC}=2\cdot \frac{I_{Right}-I_{Left}}{I_{Right}+I_{Left}},$$

where *I_Right_* and *I_Left_* represent images acquired under right-side and left-side illumination respectively [48]. The term *I_Right_* − *I_Left_* represents the difference image, while (*I_Right_* + *I_Left_*)/2 is the average image. The ratio (*I_Right_* − *I_Left_*)/(*I_Right_* + *I_Left_*) in the equation represents the intensity difference between the two illumination directions, scaled by the sum of the intensities. This scaling helps suppress spatially uniform background signals and, through normalization, enhances contrast by emphasizing directional variations caused by surface topography, phase gradients, or subtle structural features. This process highlights finer details in the specimen, making it possible to observe the intricate structure with much greater clarity. These details are often difficult to detect using conventional imaging methods, thus providing a more precise representation of the specimen’s surface.

The reason why the DPC is defined as the ratio of the difference image to the average image is that the absorption information needs to be normalized [45,48]. In the context of imaging metal workpieces, light absorption is typically uniform and negligible; this contrasts sharply with biological specimens, where absorption properties vary significantly across the specimen. In biological imaging, normalization of the absorption is essential to accurately capture phase shifts caused by structural variations. However, for metal workpieces, the minimal absorption of light makes the normalization process unnecessary. As a result, in the proposed method, the DPC is calculated simply as the difference between two images taken at consecutive time points under the illumination of different LEDs. This approach effectively isolates the phase shift information without requiring absorption normalization, thereby streamlining the process for materials with uniform light absorption characteristics. Thus, Equation (1) is simplified to Equation (2):

(2) $$I_{DPC_{weld\_seam}}=I_{Right}-I_{Left}$$

Due to the high reflectivity of the metal surface, changes in light intensity caused by different illumination angles impact the imaging results. Therefore, weighting factors are introduced into Equation (2) to adjust for the differences in light intensity caused by varying illumination angles, ensuring that the results accurately reflect the phase changes due to the specimen’s structure. The differential phase contrast with the weighting factor is shown in Equation (3):

(3) $$I_{DPC_{weld\_seam}}=w_{1}\cdot I_{Right}-w_{2}\cdot I_{Left},$$

where *w*_1_ and *w*_2_ are weighting factors that can be determined based on the inverse ratio of the average grayscale values of the two images. This approach helps mitigate the impact of intensity differences between the light sources, improving the accuracy of the phase information calculations.

### 2.2. Comparison with Existing Seam Detection Methods

Figure 3b–e compare the proposed method with current seam detection methods based on non-structured light. Figure 3c shows a typical welding seam detection system that utilizes dark-field illumination, where both LEDs are switched on during acquisition. Figure 3d illustrates a welding seam detection system employing bright-field lighting, where the optical axes of the light source and the camera are symmetrical with respect to the normal vector of the metal surface. Specular glare may occur when light from the LED is directly reflected into the camera. Bright-field illumination is generally not used in weld seam detection due to the high reflectivity of the metal surface. Additionally, there is a defocus issue in the method shown in Figure 3d because the focal plane of the camera lens is not parallel to the metal surface. To improve the stability of seam detection by capturing multiple seam features, both non-structured and structured illumination are used in the method shown in Figure 3e. As shown in the comparison, the key difference in the proposed method, depicted in Figure 3a,b, is that the phase difference of the light is also used in seam feature extraction. By converting the phase variation of the object into intensity variation in the image, the proposed method enhances the seam contrast and visualizes seam features that could not be obtained by means of traditional methods.

### 2.3. Image Processing

To demonstrate the improvement in image contrast and the versatility of the proposed method, the contrast of the seam edges acquired using different methods was compared under the same image processing conditions. Since localization of the seam position is the most important aspect of seam detection, the image contrast was evaluated by comparing the normalized gradient of the seam edges. The location of the highest gradient in the image usually indicates the seam position. For comparison, the gradient of the seam image was calculated using the Sobel operator, which is a widely used method.

We developed a software tool using LabVIEW 2023 Q1, integrated with MATLAB R2023b code, to process the images. The first step in the image processing pipeline involves applying a 2 × 2 median filter to both the left and right images. This filter aims to reduce noise and smooth the images by replacing each pixel with the median of its neighboring pixels. Specifically, for each pixel *I_Right_*_(*n,m*)_ and *I_Left_*_(*n,m*)_, the filtered values *I_Right,filtered_*_(*n,m*)_ and *I_Left,filtered_*_(*n,m*)_ are computed as the median of a 2 × 2 window centered on the pixel, which helps in preserving edge information while removing minor noise. This process is shown in Equations (4) and (5) below:

(4) $$I_{Right,filtered}(n,m)=Median(I_{Right}(n,m),I_{Right}(n+1,m),I_{Right}(n,m+1),I_{Right}(n+1,m+1)),$$

(5) $$I_{Left,filtered}(n,m)=Median(I_{Left}(n,m),I_{Left}(n+1,m),I_{Left}(n,m+1),I_{Left}(n+1,m+1)),$$

where *n* and *m* are the pixel coordinates in the vertical and horizontal directions, and Median means taking the median value of the pixels in the 2 × 2 neighborhood.

Following the filtering step, a phase image is computed by evaluating the weighted difference between the filtered left and right images. The phase difference *D*(*n,m*) at each pixel is determined by means of a linear combination of the right and left images, weighted by the respective factors *w*_1_ and *w*_2_. This is shown in Equation (6):

(6) $$D(n,m)=w_{1}\cdot I_{Right,filtered}(n,m)-w_{2}\cdot I_{left,filtered}(n,m)$$

The next step involves computing the gradient of the phase image *D*(*n,m*) using the Sobel operator. The Sobel operator applies two convolutional kernels (*S_x_* and *S_y_*). The Sobel operator is applied in both the horizontal and vertical directions to estimate the image gradient components *G_x_*(*n,m*) and *G_y_*(*n,m*), which are then combined to produce the gradient magnitude *G*(*n,m*). The horizontal (*G_x_*) and vertical (*G_y_*) derivatives of the Sobel operator are expressed in Equations (7) and (8) [49,50]:

(7) $$S_{x}=\left[\begin{array}{ccc} -1 & 0 & 1\\ -2 & 0 & 2\\ -1 & 0 & 1 \end{array}\right],\;\begin{array}{c} S_{y}=\left[\begin{array}{ccc} 1 & 2 & 1\\ 0 & 0 & 0\\ -1 & -2 & -1 \end{array}\right] \end{array},$$

(8) $$G_{x}(n,m)=D(n,m)*S_{x},\begin{array}{cc} & G_{y} \end{array}(n,m)=D(n,m)*S_{y},$$

where ∗ stands for the convolution operation.

The final gradient magnitude can be calculated using Equation (9):

(9) $$G(n,m)=\sqrt{G_{x}{\left(n,m\right)}^{2}+G_{y}{\left(n,m\right)}^{2}}$$

The final step is to normalize the gradient image by mapping the gradient values to the range [0, 1], facilitating comparisons of different images. The normalization is given in Equation (10):

(10) $$G_{normalized}(n,m)=\frac{G(n,m)-G_{\min}}{G_{\max}-G_{\min}}$$

where *G_min_* is the minimum value of the gradient image, and *G_max_* is the maximum value of the gradient image.

## 3. Experiment Results

**Figure 4:**
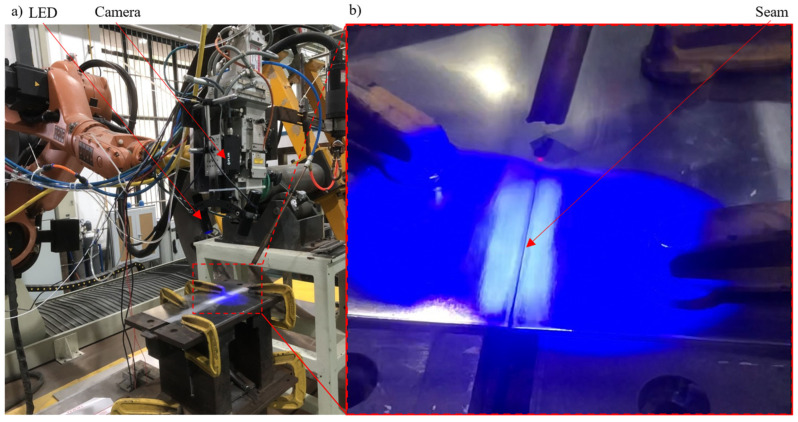
The experimental setup: (**a**) an actual photograph of the welding system; (**b**) a zoomed-in view of panel a, highlighting the workpiece.

To evaluate the flexibility and performance of the proposed seam detection system, it was mounted on a laser welding robot for the detection of weld seams of the tight square groove type. Figure 4a shows an actual photograph of the welding robot (KUKA 6-Axis Robot, Augsburg, Germany) equipped with the proposed seam tracking system. The working distance of the detection system was designed to be approximately 220 mm. The camera’s ROI was set to 200 pixels × 1000 pixels, corresponding to a field of view (FOV) of approximately 2.4 mm × 12 mm. The camera’s exposure time was set to 250 µs. The LEDs and the camera were synchronized using the control signal described in Figure 2c. The modulation frequency of the LEDs was set to approximately 500 Hz, while the image acquisition frequency was 1000 Hz, allowing a pair of DPC images to be acquired at 500 Hz. This acquisition speed was sufficient for real-time seam detection. The welding parameters are listed in Table 1. Before welding, the robot was programmed to keep the weld seam within the camera’s field of view. During welding, the detected seam position was continuously used to adjust the welding torch, ensuring precise alignment of the welding laser with the weld seam center. With a 50 mm focal length, an aperture of approximately 4.8, and a working distance of around 220 mm, the estimated depth of field of our setup was approximately 5 mm. To minimize specimen distortion during the welding process, the fixture shown in Figure 4 was carefully designed. Since the depth of focus was sufficient to maintain sharp images even with minor specimen distortion, we did not adjust the camera focus during welding. Additionally, to minimize the impact of welding light on the camera, the laser power and welding speed were carefully adjusted to control the light intensity. An argon shielding gas flow was used to stabilize the process, and the camera’s field of view was positioned 5 cm away from the welding pool to reduce direct exposure. The specimen preparation, including the base metal thickness, seam width (also called the root opening), and groove angle, followed the standards established by the American Welding Society. Detection experiments were conducted on workpieces with varying seam widths and surface flatness, simulating real-world welding scenarios. The system’s anti-noise capability was also demonstrated when welding light was present in the acquired seam images.

The proposed DPC imaging method captures images under left-side and right-side illumination separately. The combination of these two single-side images can effectively simulate images acquired under double-side illumination. This means that both single-side and double-side illumination images can be obtained through DPC imaging. Single-side and double-side dark-field illumination are common illumination schemes in current weld seam detection systems. Without loss of generality, the DPC images are compared with both single-side and double-side illumination in the following sections to demonstrate the capability of the proposed method in enhancing seam features.

### 3.1. Detection of Seams with Different Widths and Surface Flatness

Workpieces with a single-square groove were prepared using two thin aluminum plates, each with a thickness of 2 mm. Two groups of workpieces with varying seam widths, textures, and surface flatness were used to comprehensively evaluate the flexibility of the proposed detection method. One group had a seam width of approximately 0.2 mm, while the other had a seam width of 0.06 mm. The second group’s surface texture and flatness differed from those of the first group.

**Figure 5:**
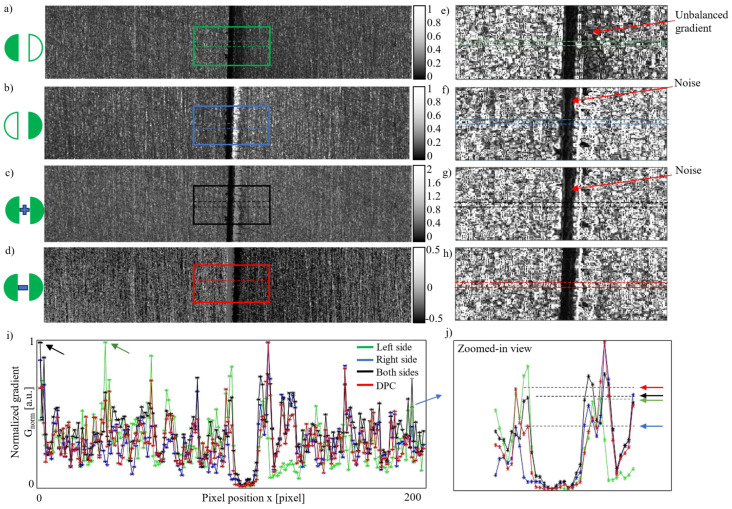
Detection results for a weld seam with a width of 0.2 mm: (**a**) a seam image acquired under left LED illumination; (**b**) a seam image acquired under right LED illumination; (**c**) the sum of the left and right images (equivalent to illumination from both sides); (**d**) the DPC image calculated as the difference between the images in (**a**,**b**) using Equation (6); (**e**–**h**) gradient images of the square areas in panels (**a**–**d**); (**i**) profiles of the average gradient intensity between the dashed lines in panels (**e**–**h**), where G_norm_ is the normalized gradient; (**j**) a zoomed-in view of (**i**).

The detection results for the first group are shown in Figure 5. Images captured under single-side illumination from the left and right sides are presented in Figure 5a and Figure 5b, respectively. Figure 5c is the combined image generated by summing the pixel values from Figure 5a,b. Specifically, for each pixel value *I_a_*(*n,m*) and *I_b_*(*n,m*) in Figure 5a,b, the corresponding pixel value *I_c_*(*n,m*) in Figure 5c was obtained as *I_c_*(*n,m*) *= I_a_*(*n,m*) + *I_b_*(*n,m*). Due to the high linearity of the camera sensor, this combined image is equivalent to an image captured under traditional double-side illumination, as shown in Figure 3b.

Figure 5d shows the DPC image generated by subtracting Figure 5b from Figure 5a using Equation (6). To visualize the seam edge contrast, the gradients within the squared regions in Figure 5a–d were calculated and are shown in Figure 5e–h based on the image processing method described in Section 2.3.

The gradient distribution on the left and right sides of the seam in Figure 5e,f is uneven: Figure 5e has a higher gradient on the left edge, while Figure 5f shows a higher gradient on the right edge. In contrast, Figure 5g,h display more balanced gradients for both seam edges. This demonstrates that double-side dark-field illumination provides better seam contrast compared to single-side illumination, which aligns with common expectations. Additionally, background noise between the seam edges is more evident in Figure 5f,g compared to Figure 5h.

To quantitatively analyze the gradient, the mean gradient values between the dashed lines in Figure 5e–h were normalized by applying Equation (10) and are plotted in Figure 5i. A zoomed-in view of the edge area is shown in Figure 5j. As indicated by the arrows in Figure 5i, edge detection based on peak gradient values failed for the left-side (green) and double-side (black) illumination images. Although the right-side illumination (blue) and differential illumination (red) successfully located the seam edge using the peak gradient, the gradient at the opposing edge under right-side illumination was much lower than that achieved with differential illumination, as shown in Figure 5j.

In conclusion, the DPC image provided the highest seam contrast in terms of the seam edge gradient and demonstrated superior performance for seam edge detection compared to the other three illumination modes.

**Figure 6:**
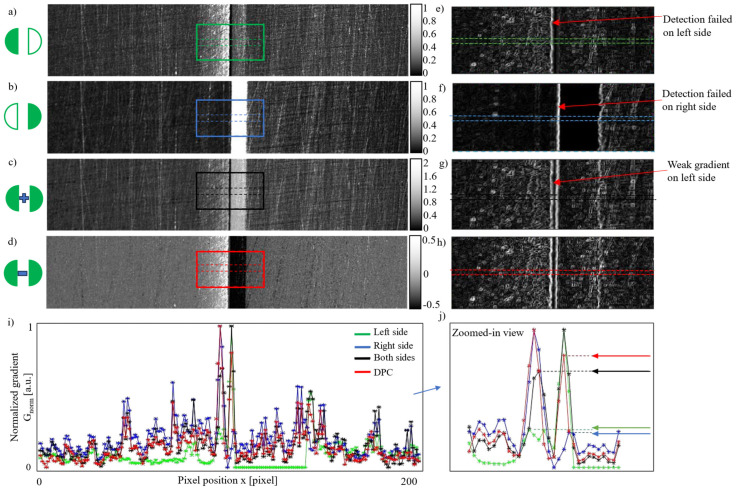
Detection results for a weld seam with a width of 0.06 mm (seam surface with non-uniform flatness): (**a**) a seam image acquired under left LED illumination; (**b**) a seam image acquired under left LED illumination; (**c**) the sum of the left and right images (equivalent to illumination from both sides); (**d**) the DPC image calculated as the difference between the images in (**a**,**b**) using Equation (6); (**e**–**h**) gradient images of the square areas in panels (**a**–**d**); (**i**) profiles of the average gradient intensity between the dashed lines in panels (**e**–**h**), where G_norm_ is the normalized gradient; (**j**) a zoomed-in view of (**i**).

For the second demonstration, a workpiece with relatively non-uniform flatness was intentionally selected to showcase the flexibility of the proposed method. The detection results are shown in Figure 6. In practice, it is challenging to ensure that all workpieces have uniform flatness. Specular reflection can still occur in seam images, even with a detection system designed based on the dark-field imaging principle.

The images acquired under single-side (left or right) illumination are shown in Figure 6a,b. Figure 6c presents the sum of Figure 6a,b. Figure 6d shows the DPC image generated by subtracting Figure 6b from Figure 6a, as defined by Equation (6). Due to the presence of specular reflection, the seam edges in Figure 6b are visually indistinguishable.

The gradient of the highlighted square areas in Figure 6a–d is shown in Figure 6e–h, calculated using the image processing method described in Section 2.3. Under single-side illumination, only one seam edge is visible, as illustrated in Figure 6e,f. Although both edges can be distinguished with double-sided illumination in Figure 6g, the left edge appears visually weaker compared to the edges in Figure 6h.

To quantitatively assess the gradient, the mean gradient values between the dashed lines in Figure 6e–h were normalized by applying Equation (10) and are plotted in Figure 6i. A zoomed-in view of the edge areas is shown in Figure 6j. All four imaging modes accurately located the seam edges based on the peak gradient values. However, the gradient values of the seam edges under differential illumination (red line) were the highest, demonstrating superior performance.

### 3.2. Anti-Noise Experiment

**Figure 7:**
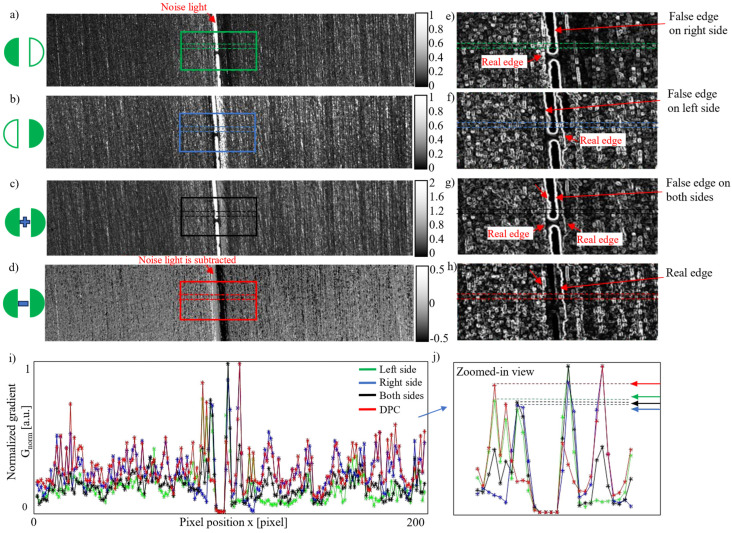
Anti-noise experiment: (**a**) a seam image acquired under left LED illumination; (**b**) a seam image acquired under right LED illumination; (**c**) the sum of the left and right images (equivalent to illumination from both sides); (**d**) the DPC image calculated as the difference between the images in (**a**,**b**) using Equation (6); (**e**–**h**) gradient images of the square areas in panels (**a**–**d**); (**i**) profiles of the average gradient intensity between the dashed lines in panels (**e**–**h**), where G_norm_ is the normalized gradient; (**j**) a zoomed-in view of panel (**i**).

To evaluate the feasibility of the proposed method, a detection experiment was conducted under welding light noise. As shown in Figure 7a–c, welding light filled the area between the seam edges, resulting in pixel saturation. The gradient images of the square areas in Figure 7a–d were processed using the same procedure described in the previous section and are presented in Figure 7e–h. In Figure 7e–g, the boundaries of the welding light noise dominate, leading to false edge detections. However, the true edges of the weld seam can be clearly distinguished in Figure 7h.

To analyze the gradient distribution more quantitatively, the mean gradient values between the dashed lines in Figure 7e–h were normalized by applying Equation (10) are plotted in Figure 7i, with a zoomed-in view shown in Figure 7j. As shown in Figure 7j, the true edges from the DPC seam image can be identified by the peak gradient values. Additionally, the normalized gradient values for the real edges in the DPC image are even higher than the false edges detected with either single-side or double-side illumination.

Since the DPC image is generated by subtracting one image from another, the welding light noise between the seam edges is effectively removed. This straightforward and efficient noise removal method is a key advantage of using the DPC approach in seam detection, enhancing its competitiveness.

### 3.3. Evaluation of Seam Detection Accuracy

In addition to comparing the seam contrast under different types of illumination, we evaluated the measurement accuracy of the proposed method. To verify the seam edge detection accuracy, the weld seam width at 30 different locations on two specimens was measured before welding using a commercial measuring apparatus (TESA-VISIO 300, Aarau, Switzerland). These measurement results were then compared with the results obtained using the proposed setup, as shown in Table 2. The mean absolute errors were less than 0.017 mm, and the maximum error was less than 0.036 mm. The standard deviation of the absolute error was less than 0.015 mm. The observed mean absolute error of 0.017 mm is likely due to the resolution of our optical setup, where each camera pixel corresponds to 0.012 mm. Overall, the proposed method demonstrated good accuracy in seam width detection under the tested conditions.

## 4. Discussion and Conclusions

Herein, we proposed a novel weld seam detection method using the DPC imaging technique for single-square groove weld seams. The DPC technique converts phase variations into intensity variations by calculating the difference between two images acquired under different illumination, yielding seam features with enhanced contrast.

By leveraging modern high-speed cameras and high-speed LED drivers, the proposed method captures DPC images at a high speed, avoiding motion artifacts. The existing vision sensors for weld seam detection primarily rely on light intensity information, discarding the valuable phase information. By retrieving the phase information, the proposed method provides unique and enhanced seam contrast, improving the seam edge gradients.

The effectiveness of the proposed method was demonstrated through side-by-side comparisons with single- and double-side illumination under varying seam sizes and surface flatness conditions. The DPC image consistently exhibited higher contrast than conventional illumination techniques. Furthermore, traditional vision-based seam detection methods struggle to locate seam positions in the presence of welding light noise. The DPC method effectively removes welding light noise by taking the difference between two consecutively acquired images under differential illumination, offering an innovative optical design solution. With its ability to enhance seam contrast and eliminate spark noise, this method enables more accurate and robust seam position detection. The weld seam detection accuracy was verified by comparing the results with a commercial measurement apparatus. The results show that the proposed method has a mean absolute error of approximately 0.017 mm. As part of future work, we plan to incorporate X-ray analysis to further evaluate the weld quality and provide a more comprehensive assessment of the proposed seam detection method’s performance.

To deal with distortion during the welding process, we will also explore using a focus adjustable camera lens to automatically correct the focus and ensure sharp images. To achieve a high frame rate and avoid motion artifacts, the FOV in the proposed method was limited to 2.4 mm × 12 mm. Future improvements could incorporate cameras with higher throughput to expand the FOV. Currently, at least two images are required to reconstruct the DPC image. Further enhancements in image contrast and robustness could be achieved by integrating multiple LEDs at varying illumination angles to expand the spatial frequency coverage. This approach would strengthen the phase contrast, enabling more reliable seam detection under diverse welding conditions. To avoid motion artifacts and further increase the welding speed, a high-power LED light source can be employed, and the exposure time can be further reduced to minimize the time lag between the captures of two consecutive images.

The system was designed under the dark-field imaging principle to accommodate the high reflectivity of aluminum workpieces. However, the DPC-based method can be seamlessly adapted to bright-field seam detection systems for non-metallic or less reflective workpieces. Additionally, it can be extended to other seam detection techniques that utilize structured light sources. Therefore, the proposed imaging technique has strong potential for integration into the commercial vision sensors used in automated welding systems, such as robotic-arm welding machines for laser welding. Enhancing the capability of existing vision sensors to detect narrow-gap weld seams could significantly improve welding accuracy and reliability in industrial applications.

In conclusion, the proposed method leverages the DPC technique to computationally recover phase information, providing unique and improved seam contrast. By directly removing welding light noise through differential image processing, this approach offers a powerful and innovative solution for accurate and robust weld seam detection.

## Figures and Tables

**Table 1:** Welding parameters.

Item	Description
Weld groove type	Tight single-square groove (seam width: ~0.06 [mm], ~0.2 [mm]; groove angle: 0°)
Seam type	Straight line
Material of the workpiece	Aluminum plate (type: 6061; 2 [mm] thickness)
Laser power	1.5 [kW]
Welding speed	1 [m/min]

**Table 2:** Measurement accuracy evaluation.

Item	Mean Absolute Error [mm]	Maximum Error [mm]	Standard Deviation [mm]
Specimen with 0.2 mm seam width	0.016	0.036	0.015
Specimen with 0.06 mm seam width	0.017	0.036	0.014

## Data Availability

The original contributions presented in this study are included in the article. Further inquiries can be directed to the corresponding author.

## Nomenclature

The following nomenclature is used in this manuscript:

DPC	Differential phase contrast
OCT	Optical coherence tomography
LED	Light-emitting diode
GAN	Generative adversarial network
AFFNet	Attention-Enhanced Feature Fusion Network
PCB	Printed circuit board
ROI	Region of interest
FOV	Field of view
